# Comprehensive analysis of pyroptotic gene prognostic signatures associated with tumor immune microenvironment and genomic mutation in breast cancer

**DOI:** 10.3389/fimmu.2022.933779

**Published:** 2022-08-25

**Authors:** Hongfei Zhang, Xiafei Yu, Junzhe Yang, Gao He, Xiaoqiang Zhang, Xian Wu, Li Shen, Yi Zhou, Xuyu Cheng, Xiaoan Liu, Yanhui Zhu

**Affiliations:** Department of Breast Surgery, The First Affiliated Hospital of Nanjing Medical University, Nanjing, China

**Keywords:** breast cancer, pyroptosis, pyroptotic gene prognostic signatures, tumor immune microenvironment, genomic mutation, protein ubiquitination, drug sensitivity, *in vitro* experiment targeted *CCL5*

## Abstract

**Background:**

Breast cancer is becoming a tumor with the highest morbidity rate, and inflammation-induced cell death namely pyroptosis reportedly plays dual roles in cancers. However, the specific mechanism between pyroptosis and the clinical prognosis of breast cancer patients is indistinct. Hence, novel pyroptosis-related biomarkers and their contributing factors deserve further exploration to predict the prognosis in breast cancer.

**Methods:**

Pearson’s correlation analysis, and univariate and multivariate Cox regression analysis were utilized to obtain six optimal pyroptosis-related gene prognostic signatures (Pyro-GPS). The risk score of each breast cancer patient was calculated. Next, a Pyro-GPS risk model was constructed and verified in TCGA cohort (n=1,089) and GSE20711 cohort (n=88). Then analyses of immune microenvironment, genomic variation, functional enrichment, drug response and clinicopathologic feature stratification associated with the risk score of Pyro-GPS were performed. Subsequently, a nomogram based on the risk score and several significant clinicopathologic features was established. Ultimately, we further investigated the role of *CCL5* in the biological behavior of MDA-MB-231 cell line.

**Results:**

The low-risk breast cancer patients have better survival outcomes than the high-risk patients. The low-risk patients also show higher immune cell infiltration levels and immune-oncology target expression levels. There is no significant difference in genetic variation between the two risk groups, but the frequency of gene mutations varies. Functional enrichment analysis shows that the low-risk patients are prominently correlated with immune-related pathways, whereas the high-risk patients are enriched in cell cycle, ubiquitination, mismatch repair, homologous recombination and biosynthesis-related pathways. Pyro-GPS is positively correlated with the IC50 of anti-tumor drugs. Furthermore, comprehensive analyses based on risk score and clinicopathological features were performed to predict the prognosis of breast cancer patients. Additionally, *in vitro* experiments confirmed that breast cancer cells with high expression of *CCL5* had weaker proliferation, invasion and metastasis abilities as well as stronger apoptosis and cell cycle arrest abilities.

**Conclusions:**

The risk score of Pyro-GPS can serve as a promising hallmark to predict the prognosis of BRCA patients. Risk score evaluation may assist to acquire relevant information of tumor immune microenvironment, genomic mutation status, functional pathways and drug sensitivity, and thus provide more effective personalized strategies.

## Introduction

The latest data on the global burden of cancers in 2020 released by the World Health Organization show that the number of new cases of breast cancer in 2020 reached 2.26 million, accounting for about 11.7% of all cancers and surpassing the 2.2 million cases of lung cancer (11.4%) (1). Thus, breast cancer is becoming the cancer with the highest incidence in the world. At present, molecular subtype, tumor size, lymph node status, and the existence of metastasis are used to evaluate the survival outcome and select relevant treatment measures for breast cancer patients. Surgical therapy, chemotherapy, radiotherapy, Her-2 targeted therapy and endocrine therapy are extensively applied in clinical practice (2). However, traditional prediction methods and therapeutic strategies can no longer meet our needs along with the increasing demand for accuracy in prognosis prediction and individualized treatment as well as the prominent heterogeneity for breast cancer whether in primary sites or in metastatic sites (3–5). Therefore, the identification of novel sensitive tumor prognostic biomarkers deserves intensive investigation for more advanced diagnosis and treatments.

Currently, cell pyroptosis and its dual anti-tumor and pro-tumor effects have aroused wide concern (6, 7), prompting us to focus on the significant associations of pyroptosis with the treatment and prognosis of breast cancer. Pyroptosis, an inflammatory form of programmed cell death (8), relies strongly on the gasdermin protein family to form plasma membrane pores, usually (but not always) resulting in inflammatory caspase activation (9). Specifically, cell pyroptosis occurs through four pathways: 1) classic pathway (Caspase-1 mediated) (10), 2) non-classic pathway (Caspase-4/5/11 mediated) (10, 11), 3) Caspase-3/8 mediated pathway (12, 13), and 4) granzyme-mediated pathway (13, 14). Among them, the classic pathway relies on the assembly of inflammasomes (15) composed of cytoplasmic pattern recognition receptors (PRRs), apoptosis-associated speck-like protein containing a CARD (ASC) and caspase-1. Firstly, PRRs including NLRP1, NLRP3, NLRC4 (IPAF), AIM2 and Pyrin recognize foreign pathogen-associated molecular patterns and endogenous damage-associated molecular patterns, and then activate precursor caspase-1 directly or indirectly through the adaptor protein ASC. After that, mature caspase-1 can hydrolyze GSDMD to generate N-GSDMD, resulting in plasma membrane pore formation, cell swelling and even osmotic lysis. Meanwhile, pro-IL-1β and pro-IL-18 can be processed by caspase-1 to form mature IL-1β and IL-18, which are then released to produce inflammatory response.

Much evidence shows that pyroptosis is closely associated with diverse tumors (e.g. melanoma, breast cancer, and tumors of digestive, respiratory, hematological and reproductive systems), regulating the proliferation, invasion and metastasis of tumor cells through some non-coding RNAs and other molecules (16). Meanwhile, pyroptosis plays distinct and sometimes conflicting roles in the occurrence and development of tumors (17). On the one hand, inflammasome activation that forms an inflammation environment may suppress antitumor immunity and tumor cell death, and even facilitate tumor cell proliferation and angiogenesis. On the other hand, pyroptosis can inhibit tumorigenesis and progression, inducing pyroptosis of tumor cells, and can serve as a potential therapeutic strategy (18). For instance, BRAF-MEK inhibition can activate GSDME-dependent pyroptosis to enhance the immune recognition of melanoma cells, including an increase in CD4+ T cell and CD8+ T cell infiltration and a decrease in myeloid-derived suppressor cells (MDSCs) and tumor-associated macrophages (19–21). Chimeric antigen receptor (CAR) T cells can activate the caspase 3–GSDME pathway *via* releasing perforin and granzyme B to trigger pyroptosis in B leukemic cells (22). In addition, the downregulation of GSDMD can activate ERK1/2, STAT3 and PI3K/AKT signaling pathways, and increase the expression of cell cycle-related proteins (Cyclin A2 and CDK2) to accelerate S/G2 phase cell transition in gastric cancer (23). Moreover, GSDMD deficiency will alleviate the cytolytic ability of CD8+ T cells (24). However, GSDMD as a pyroptosis executive protein is more highly expressed in non-small cell lung cancer (NSCLC) than para-cancer tissues, and knockdown of GSDMD can attenuate the EGFR/Akt signaling pathway to restrict tumor growth in NSCLC (25). Recently, most studies emphasize the tumor suppression function and anti-tumor immune responses of pyroptosis. Moreover, therapeutic regimens such as chemotherapy, radiotherapy, targeted therapy and immune therapy can induce cancer pyroptosis to exert local and systemic anti-tumor immune response (26). Hence, pyroptosis may be intricately associated with tumors through the alteration of the tumor immune microenvironment (TIME). Then clinical modeling based on pyroptosis-related biomarkers to predict the TIME and immunotherapeutic efficacy of breast cancer deserves further exploration.

In our work, the prognostic value of pyroptosis-related genes was proved by a series of bioinformatic and statistical analyses based on the data curated from The Cancer Genome Atlas (TCGA) and Gene-Expression Omnibus (GEO). Then six pyroptosis- related gene prognostic signatures (Pyro-GPS) were identified to calculate the risk score for each breast cancer patient. After that, the breast cancer patients were stratified by the median risk score into a high-risk group and a low-risk group. Patients in the low-risk group had more favorable survival than those in the high-risk group. To elucidate possible mechanism, we further explored the variations of the risk score in the aspect of immune cell infiltration, immune-oncology targets, genomic variations, differentially expressed genes (DEGs), pathway enrichment status and anti-tumor drug response. Eventually, a nomogram model based on the risk score and other clinicopathological features was constructed to predict the overall survival (OS) of the breast cancer patients. What’s more, one of Pyro-GPS, *CCL5* as hub gene was screened out to proceed *in vitro* validation.

## Materials and methods

### Data acquisition

Transcriptomic data (including RNA-seq, gene chip and small RNA-seq), genomic data (somatic mutation and copy number variation) and corresponding clinicopathological features (e.g. survival time, prognosis, chemotherapy, age, gender, tumor stage, T stage, M stage, N stage and PAM50 intrinsic subtypes) of 1089 breast cancer patients were derived from TCGA (https://portal.gdc.cancer.gov/). Moreover, 21 pyroptosis genes were identified based on previous publications, and the expression matrices of 6 optimal prognosis-related genes (*CD2*, *CCL5*, *KLRB1*, *CD74*, *NLRC4* and *ZNF683*) were extracted from TCGA. We obtained the profiles for drug sensitivity data as half maximal inhibitory concentration (IC50) from CellMiner (https://discover.nci.nih.gov/cellminer/home.do). In addition, the RNA-seq data and clinical information of the external validation cohort involving 88 breast cancer cases were downloaded from GEO (https://www.ncbi.nlm.nih.gov/geo/, ID: GSE20711).

### Analysis process

Firstly, gene expression and transcriptome data in the format “FPKM” was downloaded from TCGA database. Secondly, a set of genes strongly correlated with 21 pyroptosis genes were obtained by Pearson’s correlation analysis then univariate and multivariate Cox regression analysis produced six genes associated with prognosis (Pyro-GPS). Next, a Pyro-GPS risk prognostic model was constructed *via* TCGA training set and verified internally *via* TCGA testing set. Then, we continued investigating the possible causes of prognosis discrepancies in several aspects such as immune microenvironment, genomic variation, functional enrichment, drug response and clinicopathologic feature. In addition, external validation was performed through data from GEO database and nomogram was established to predict survival time for breast cancer patients. Eventually, *CCL5* as hub gene was selected for *in vitro* validation, including EdU (5-Ethynyl-2’-deoxyuridine), CCK8 (Cell Counting Kit-8), colony formation assay, flow cytometry and transwell assay.

### Construction and validation of the pyroptosis-related prognostic model

The 1089 breast cancer patients from TCGA were randomly separated into a training cohort and a testing cohort using the R package Caret. Pearson’s correlation analysis based on the 21 pyroptosis genes above was performed to acquire 161 pyroptosis-related genes. Then 6 optimal pyroptotic genes correlated with prognosis were identified through univariate and multivariate Cox regression analyses. The risk score of every patient was calculated as: Risk score= $\sum_{i}^{6}{\text{Coef}}_{i}*X_{i}$ (*Coef_i_*: coefficient, *X_i_*: expression level of each prognosis-related gene). Thus, a Pyro-GPS model for the breast cancer patients was constructed in the training cohort and preliminarily verified in the testing cohort and an external validation cohort GSE20711.

### Tumor immune microenvironment in risk model

With the R package Gsva, single sample gene set enrichment analysis (ssGSEA) was implemented to quantify the relative abundance of 28 immune-cell types by using enrichment scores based on the mRNA expression level of breast cancer tissues. We also applied the package Estimate to calculate the Stromal score, Immune score, ESTIMATE score, and Tumor purity and to draw clustering heat maps based on the transcriptome expression profiles of breast cancer. The data on tumor- immune infiltration was obtained from Tumor Immune Estimation Resource (TIMER 2.0) (http://timer.comp-genomics.org/). Algorithms such as XCELL, CIBERSORT and MCP- counter were used to evaluate the proportions of infiltrated immune cells on TIMER 2.0.

### Gene mutation status in risk model

We extracted somatic mutation data from the Genomic Data Commons (GDC) data portal (https://portal.gdc.cancer.gov/) and drew oncoplots with the R package ComplexHeatmap. The mutation annotation format of somatic variants was downloaded, and multiple analysis modules were visualized on the R package Maftools. Besides, tumor mutation burden was identified as the number of detected somatic mutations, including gene-coding errors, base substitutions, and insertions or deletions per million bases. Copy number variation (CNV) as a structural variation of DNA represents amplification and deletion of DNA fragments larger than 1 KB in length.

### Differentially expressed genes and function enrichment analysis

Based on RNA-seq data, DEGs were identified by the R packages edgeR and limma with fold change (FC) =2 and false discovery rate (FDR)<0.05. Function enrichment analyses including gene set enrichment analysis (GSEA) and gene set variation analysis (GSVA) were performed to explore the differences in biofunctions and signaling pathways. The package clusterProfiler was used for functional annotation and the gene set files were extracted from MsigDB (https://www.gsea-msigdb.org). Besides, gene annotations were finished with Gene Oncology (GO), hallmark, and Kyoto Encyclopedia of Genes and Genomes (KEGG) pathway analyses. GO was further divided into biological process (BP), cellular component (CC) and molecular function (MF).

### Pyro-GPS related drug sensitivity

Three Pyro-GPSs (*CD2*, *ZNF683* and *KLRB1*) and corresponding antitumor drug sensitivity in cancer cell lines were available from Genomics of Drug Sensitivity in Cancer (http://www.cancerrxgene.org/downloads). In addition, IC50, namely the concentration of an antitumor drug that is required to inhibit 50% cancer cells, was used to represent drug sensitivity.

### Validation the function of CCL5 *in vitro* experiments

#### Cell culture and transfection

MDA-MB-231 cells were cultured in 10% fetal bovine serum in a humidified atmosphere of 5% CO2 at 37°C and cell transfection was accomplished by using Lipofectamine^®^ 3000. Subsequently, the CCL5 protein expression level was detected by western blot analysis to confirm whether the cell line was constructed successfully.

#### Western blot analysis

Western blot was a multistep procedure including a) proteins extracted from MDA-MB-231 cells *via* lysis buffer after PBS washing, b) proteins separated by sodium dodecyl sulphate (SDS) polyacrylamide (10%) gel electrophoresis and transferred onto polyvinylidene fluoride (PVDF) membranes, c) proteins immobilized and blocked on the membrane with 5% nonfat dry milk, d) membranes incubated with primary antibody of rabbit polyclonal anti-CCL5 (1:1,000), e) membranes washed and incubated with secondary antibody (1:4,000) conjugated with labelled fluorescent molecule, f) labeled proteins visualized by the enhanced chemiluminescence kit; g) signal intensity of CCL5 protein bands collected by a digital imaging computer software.

#### Cell proliferation and colony forming assay

Cell viability was measured through Cell Counting Kit-8 (CCK8) and EdU (5-Ethynyl-2’-deoxyuridine) assays. Briefly, 10 µl CCK8 solution (DOJINDO) was respectively added to each well/2×10^3 cells in a 96-well plate, and the plate was further incubated at 37°C for 2 hours. The absorbance of each well was measured at a wavelength of 450 nm(OD450) with a microplate reader and proliferation rates were calculated. Additional, cells were re-cultured in 96-well plates, then 10 μM EdU (BeyoClick™) was added into the medium and continued two-hour incubation. Next these cells were stained with 4’,6-diamidino-2-phenylindole (DAPI) and inverted fluorescence microscope was used to acquire the images. As for cell colony formation assay, 8×10^2 cells were planted and cultured in each well of a 6-well plate, then they were fixed with 4% paraformaldehyde for 15 min and stained with 0.1% crystal violet for 20 min at room temperature. Lastly, the number of clones was imaged with a light microscope at ×40 magnification.

#### Flow cytometry of cell cycle and apoptosis

As we all know, the DNA content of different cell cycle phases is different (cells in G0/G1 phase is diploid (2N), cells in G2/M phase is 4N, while cells in S phase is between diploid and tetraploid). Thus, flow cytometry could directly reflect the amount of DNA content in cells *via* detecting the fluorescence intensity of propidium iodide (PI) bound to DNA. Firstly, the cells were pretreated with ice-cold 70% ethanol and were washed with PBS, then a cell cycle staining kit (LiankeBio) was used. 10 µl Rnase A was added to cell resuspension solution for 5 min at room temperature to digest RNA, then 10 µl PI was added to bind to DNA for 30 min at room temperature. Finally, flow cytometer detected DNA content and the percentage of each phase could be calculated by special software. In addition, apoptotic analysis was performed through a Annexin V-FITC/PI Apoptosis Assay kit and cells were double stained with Annexin V-FITC and PI for 15 min at room temperature. Since Annexin V-FITC could mark early apoptotic cells and PI could label cells in the middle and late stage of apoptosis, then cell apoptosis condition could be detected by flow cytometry.

#### Cell invasion and migration assay

Transwell assay was utilized to detect cell invasion and migration ability. Firstly, eight-micrometer pore-size transwell filters (Millipore) were put in 24-well plate and a concentration of 1×10^4 cells/well were seeded onto the filters. Then 200 μL FBS-free medium was added into the upper chamber while the lower chamber was filled with 600 μL of medium with 10% FBS. After cultivation of 48 hours at 37°C, these invasive and metastatic cells in the lower side of the filter were determined *via* crystal violet staining and were counted at ×400 magnification with a light microscope.

### Statistical analyses

All the statistical analyses were performed on R Version 4.0.1. If not specifically mentioned, P< 0.05 was considered as a statistical difference. R packages Survival and Survminer were implemented for Kaplan-Meier survival analysis to visualize the survival differences. Log-rank test and Gehan-Breslow-Wilcoxon test were carried out to estimate the statistical significance of survival differences between groups. Univariate and multivariate Cox regression analyses were performed to assess the independent prognostic value of Pyro-GPS for breast cancer patients. We calculated Pearson’s correlation coefficients to confirm the correlation between two variables. We applied the waterfall function of the package Maftools to visualize the mutation landscape. The predictive accuracy of the risk score and other clinicopathological features was estimated using receiver’s operating characteristic (ROC) curves and the area under the ROC curve (AUC). We established a nomogram including clinical features and risk score *via* the package Rms, and the calibration plots illustrated the prediction accuracy.

## Results

### Identification of pyroptosis-related genes in breast cancer patients

**Figure 1:**
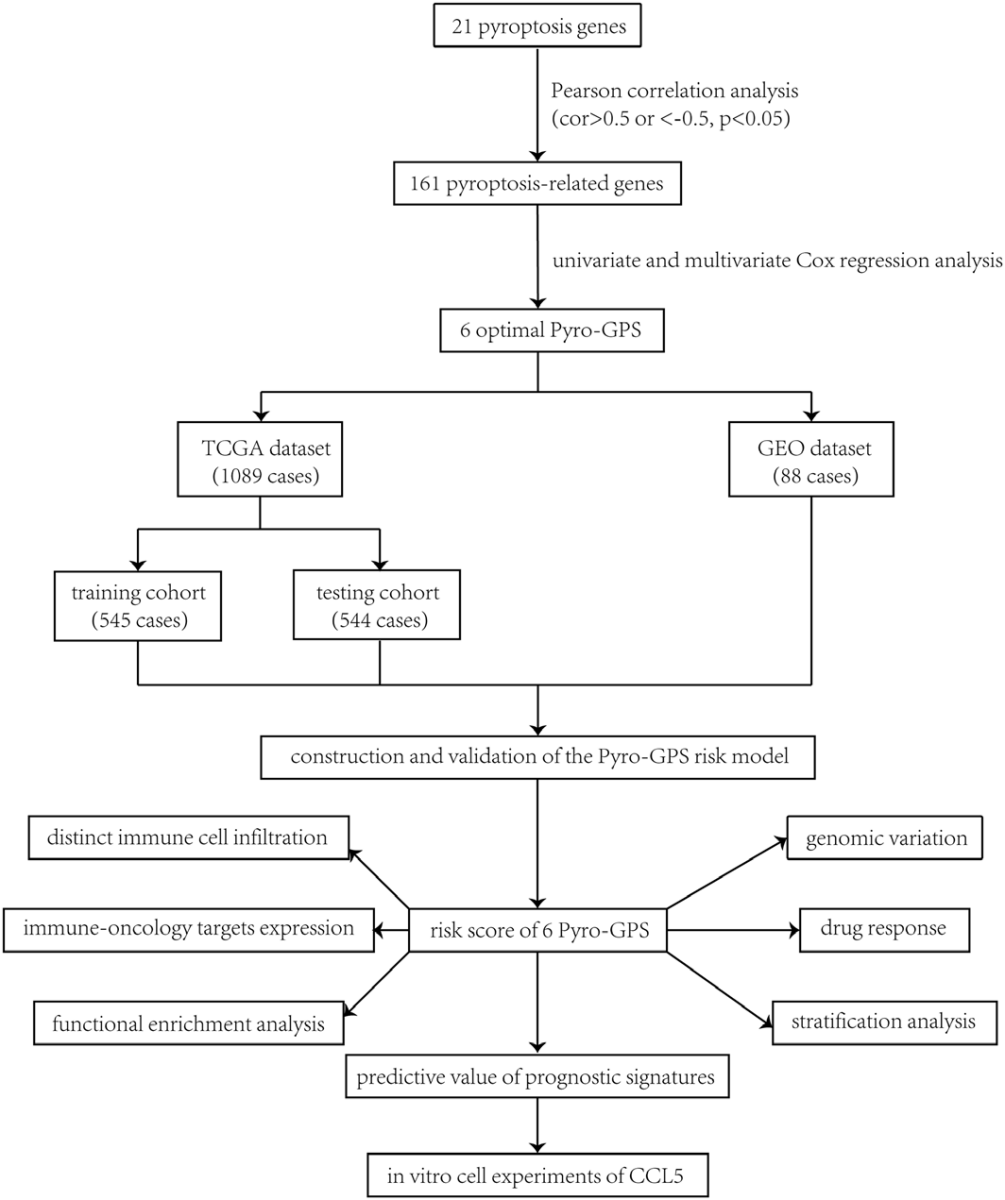
Study flowchart.

The study procedure was illustrated in **Figure 1**. Firstly, a total of 161 pyroptosis-related genes were extracted from Pearson’s correlation analysis (| Pearson R| > 0.5 and p< 0.05) based on 21 pyroptosis genes. According to the univariate and multivariate Cox regression analyses, 6 optimal survival-related genes (*CD2*, *CCL5*, *KLRB1*, *CD74*, *NLRC4* and *ZNF683*) that met the criterion of p< 0.2 were retained for further analysis (**Supplementary Figure 1A**). Among them, *CD2* and *NLRC4* were considered as risky factors with hazard ratio (HR) >1, whereas the other 4 genes were defined as protective factors with HR<1. Afterward, 1,089 breast cancer patients identified from TCGA were randomly assigned to the training cohort (n =545) and the testing cohort (n =544) at a nearly 1:1 ratio.

### Construction and validation of the Pyro-GPS model in the TCGA cohort

**Figure 2:**
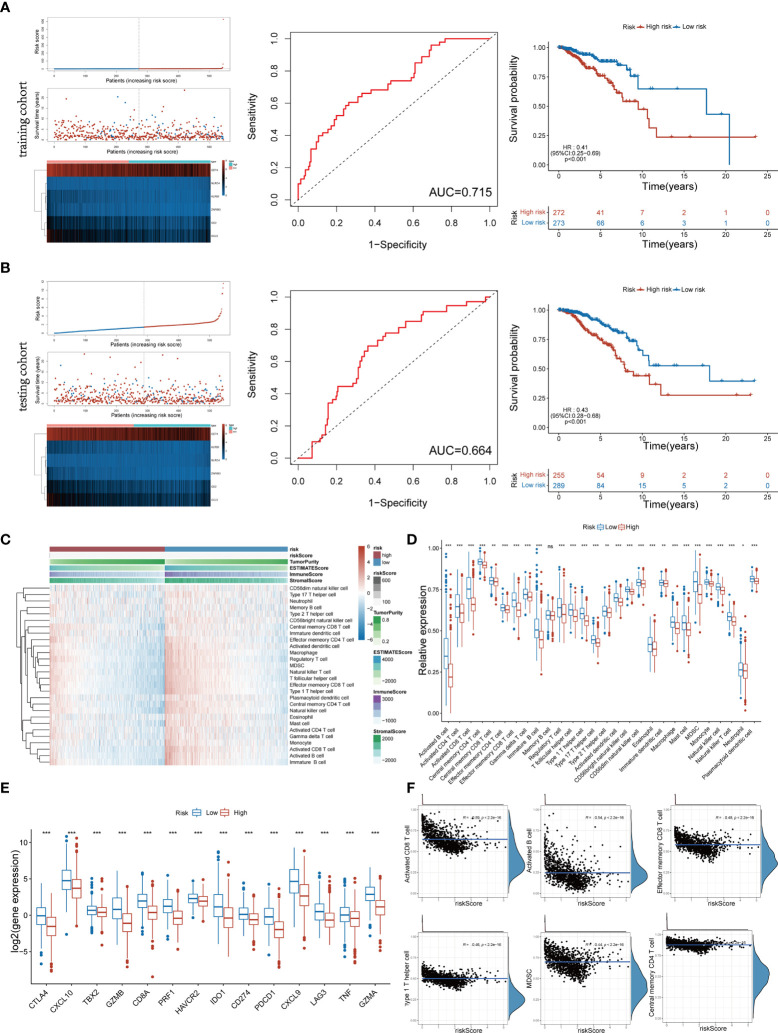
Distribution of risk scores and survival status, heatmap of six Pyro-GPS, and Kaplan–Meier curves of OS for BRCA patients in TCGA training cohort **(A)** and testing cohort **(B)**. **(C)** Heatmap of correlations between risk scores with ImmuneScore, StromalScore, ESTIMATEScore, Tumor Purity and 28 immune cell types. **(D)** The infiltration levels of 28 immune cell types in high-/low-risk subtypes. *p < 0.05, **p < 0.01 and ***p < 0.001. **(E)** The expression levels of 14 immune-oncology targets in high-/low-risk subtypes. **(F)** The relevance between infiltration of activated CD8 T cell, activated B cell, effector memory CD8 T cell, type 1 T helper cell, MDSC and central memory CD4 T cell with risk score. Pyro-GPS, pyroptosis-related genes prognostic signatures; OS, overall survival; BRCA, breast cancer; TCGA, The Cancer Genome Atlas. ns, no significance.

The above 6 genes associated with overall survival (OS) were defined as Pyro-GPS to construct a prognostic model. The risk score of each patient based on the Pyro-GPS was calculated as follows: risk score=(0.106* *CD2* exp.)+(-0.0151* *CCL5* exp.)+(-0.482* *KLRB1* exp.)+(-0.00124* *CD74* exp.)+(0.898* *NLRC4* exp.)+(-0.330* *ZNF683* exp.). According this formula, 545 patients in the training cohort and 544 patients in the testing cohort were divided into low-risk and high-risk groups respectively. Besides, the distributions of risk scores and survival time of the two cohorts were plotted in **Figures 2A, B**. The heatmaps showed that the expression levels of the protective Pyro-GPSs (*CCL5*, *KLRB1*, *CD74*, and *ZNF683*) were downregulated and the risky Pyro-GPSs (*CD2*, and *NLRC4*) were upregulated with the increasing risk score. Then ROC analysis was performed to assess the sensitivity and specificity of the prognostic model, and the AUC for the six Pyro-GPSs was 0.715 in the training cohort and 0.664 in the testing cohort. Moreover, the Kaplan–Meier survival curves disclosed that patients in the low-risk group had survival advantages and patients in the high-risk group had poor prognosis (p< 0.01 in both cohorts). Therefore, the significant difference in the survival rate between the risk groups also confirmed the predictive value of the model constructed by Pyro-GPS.

### Association of Pyro-GPS with distinct immune cell infiltration and immune-oncology targets

To identify the relationship between Pyro-GPS and TIME of BRCA, we investigated the immune infiltrate levels in the high- and low-risk groups. The boxplot in **Figure 2D** compared the proportions of 28 distinct immune cells in the two groups, where the low-risk group had a higher infiltration level excluding memory B cell compared with the high-risk group. Significant differences were found in activated B cell, activated CD4 and CD8 T cells, central memory CD4 T cell, effector memory CD4 T and CD8 T cells, gamma delta T cell, immature B cell, regulatory T cell, T follicular helper cell, type 1 and type 17 T helper cells, activated and plasmacytoid dendritic cells, CD56bright and CD56dim natural killer cells, eosinophil, macrophage, mast cell, MDSC, monocyte, natural killer cell, and natural killer T cell (p<0.001). In addition, the heatmap in **Figure 2C** visualized the expression levels of 28 diverse immune cells and displayed the features of Risk score, ImmuneScore, StromalScore, ESTIMATEScore and Tumor purity in both high-risk and low-risk groups. Moreover, principal component analysis (PCA) showed that risk score was significantly and negatively correlated with 21 immune infiltrating cell types under the qualifications of p< 0.001 and |Person R| > 0.2. Then we selected and displayed a part in **Figure 2F**, and showed the remaining part in **Supplementary Figure 1B**. Subsequently, we compared the expression levels of immune-oncology targets between the two groups to ascertain their relevance with Pyro-GPS. Results indicated that the low-risk group highly expressed CTLA4, CXCL10, TBX2, GZMB, CD8A, PRF1, HAVCR2, IDO1, CD274, PDCD1, CXCL9, LAG3, TNF and GZMA than the high-risk group (with conspicuous significance, p<0.001) (**Figure 2E**). Thereby, due to the abundant TIME in the low-risk group, immunotherapy may respond better.

### Risk score correlated with genomic mutation status

**Figure 3:**
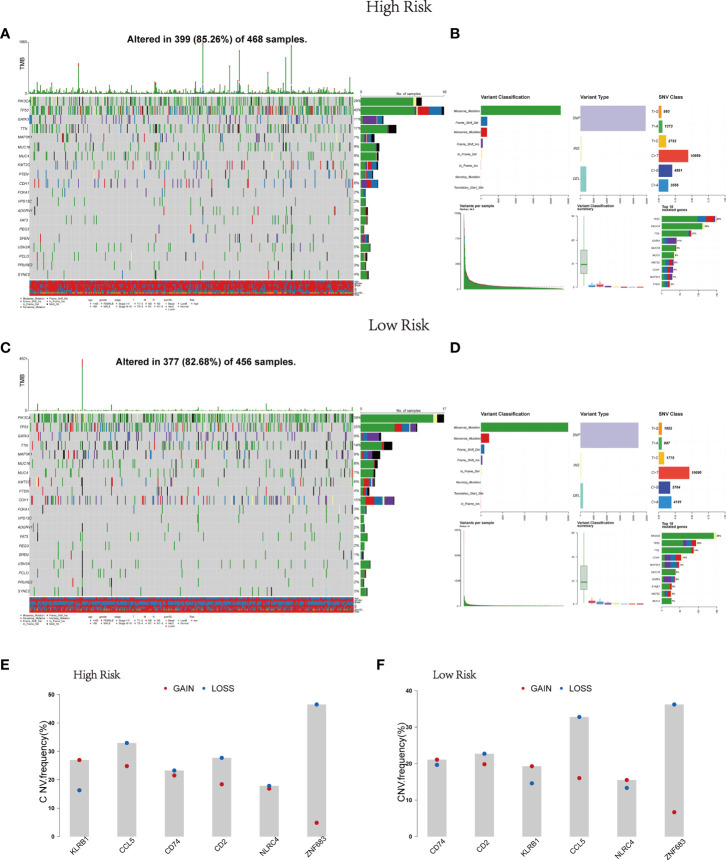
Landscape of mutation profiles in BRCA samples. Waterfall plots of 20 genes mutation information and bar plots of TMB in high-risk subtype **(A)** and low-risk subtype **(C)**. Summary of the mutation information with statistical calculations in high-risk subtype **(B)** and low-risk subtype **(D)**. **(E, F)** The difference of CNV for six Pyro-GPS in high- and low-risk subtypes. BRCA, breast cancer; TMB, tumor mutational burden; CNV, copy number variation; Pyro-GPS, pyroptosis-related genes prognostic signature.

Afterwards, the gene mutation status in the risk model was further analyzed through mutation profiles of the breast cancer patients obtained from TCGA. Gene alteration occurred in 399 (85.26%) of 468 samples in the high risk group and in 377 (82.68%) of 456 samples in the low risk group (**Figures 3A, C**). Mutation information of the 20 genes was visualized in waterfall plots. Among them, *TP53* possessed the highest mutation frequency, accounting for 40% in the high-risk model, but *PIK3CA* had the highest mutation frequency in the low-risk model (38%). Meanwhile, the tumor mutational burden levels of the two risk groups were exhibited in the upper bar plots. Moreover, these mutations were further divided into diverse categories (**Figures 3B, D**). Then similar results in the two groups were discovered that missense mutation occupied the main portion, single-nucleotide polymorphism (SNP) occurred more frequently than insertion (INS) or deletion (DEL), and C>T was the most common type of single nucleotide variant (SNV). **Figure 3** also depicted the number of variants in each sample, variant classification summary and the top 10 mutated genes in the two groups. **Figures 3E, F** displayed the CNV status of the six Pyro-GPSs. Among them, the frequency of CNV in *NLRC4* was slightly different between the high- and low-risk groups.

### Interactions of risk score with DEGs and functional enrichment analysis

**Figure 4:**
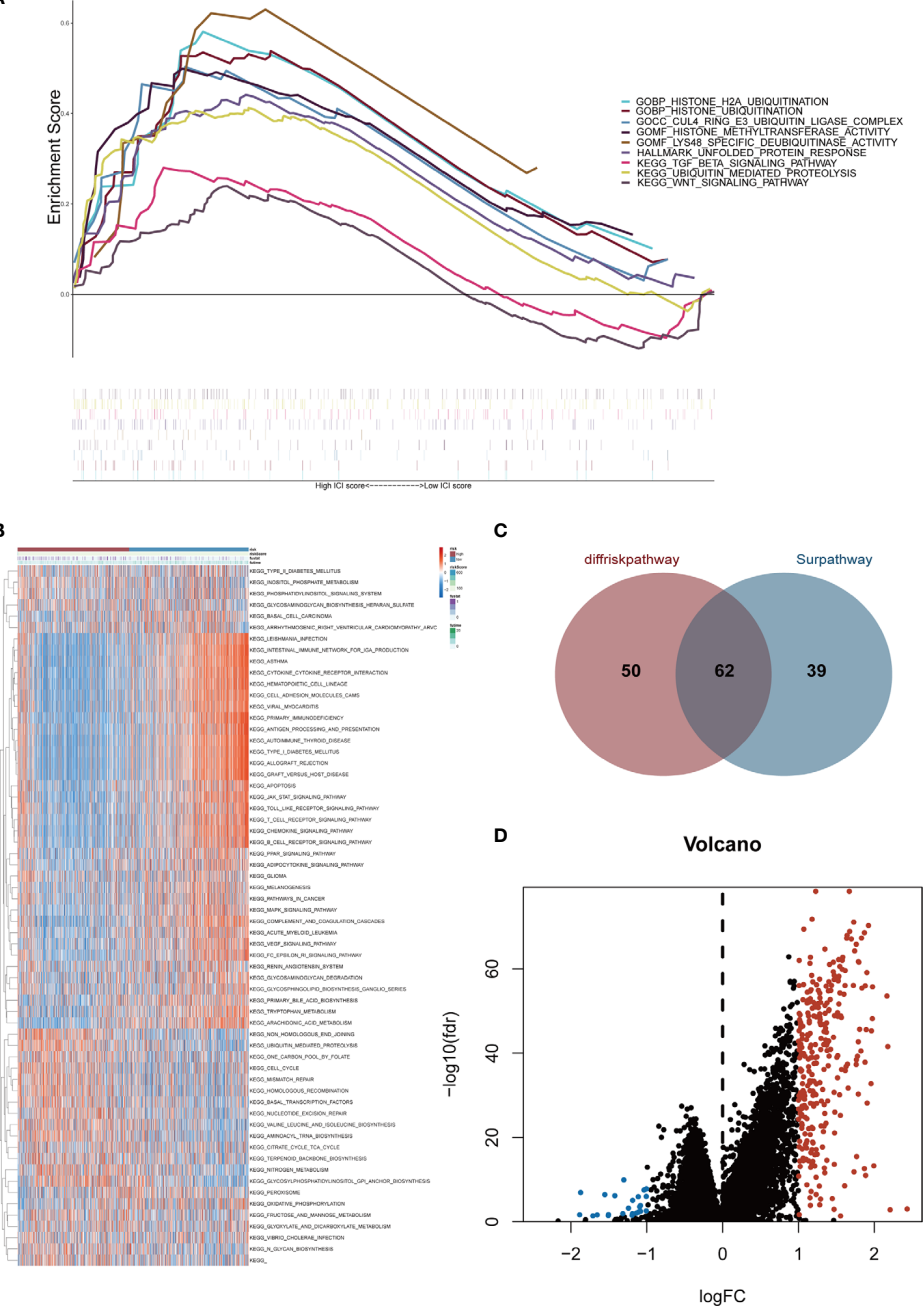
**(A)** Gene set enrichment analysis (GSEA) illustrated the functional enrichment and pathway enrichment in the high-risk subgroup. **(B)** Gene set variation analysis (GSVA) enumerated difference of enriched pathways between high-/low-risk subtypes. **(C)** Venn diagram of quantity statistics for KEGG pathways correlated with risk score and survival time. **(D)** Volcano plot depicted differential expression genes in high-/low-risk subtypes (|logFC| >1 and FDR<0.05). KEGG, Kyoto Encyclopedia of Genes and Genomes.

Gene expression conditions in the high- and low-risk groups were demonstrated in the heatmap (**Supplementary Figure 2**). The volcano plot displayed 324 DEGs, including 300 up-regulated genes (red dots) and 24 down-regulated genes (blue dots) with the cut-off criteria of |logFC| >1 and FDR<0.05 (**Figure 4D**). After that, GSEA was performed based on DEGs. Then we found enrichment signaling pathways were significantly correlated with protein ubiquitination, deubiquitination, TGF-β and Wnt (**Figure 4A**). Moreover, GSVA proved that sixty-two KEGG pathways were remarkably different between the two groups (**Figure 4B**). Among them, the high-risk group was heavily enriched in cell cycle, ubiquitination, mismatch repair, homologous recombination and biosynthesis-related pathways. The low-risk group was remarkably correlated with infection, immune response, immune rejection, autoimmune disease, apoptosis, immune globulin, cytokine, chemokine, complement and other signaling pathways (e.g. T cell receptor, B cell receptor, Toll-like receptor, JAK-STAT, PPAR, MAPK, VEGF pathways). These analyses suggest that the risk score of the six Pyro-GPSs is associated with TIME, gene variation and protein ubiquitination. Besides, the Venn diagram in **Figure 4C** shows that 112 and 101 KEGG pathways are related with the risk score and survival time respectively. Among them, 62 common pathways are associated with both risk score and survival time. Details about the pathways can be found in the **Supplementary Data**.

### Potential therapeutic value of risk score

**Figure 5:**
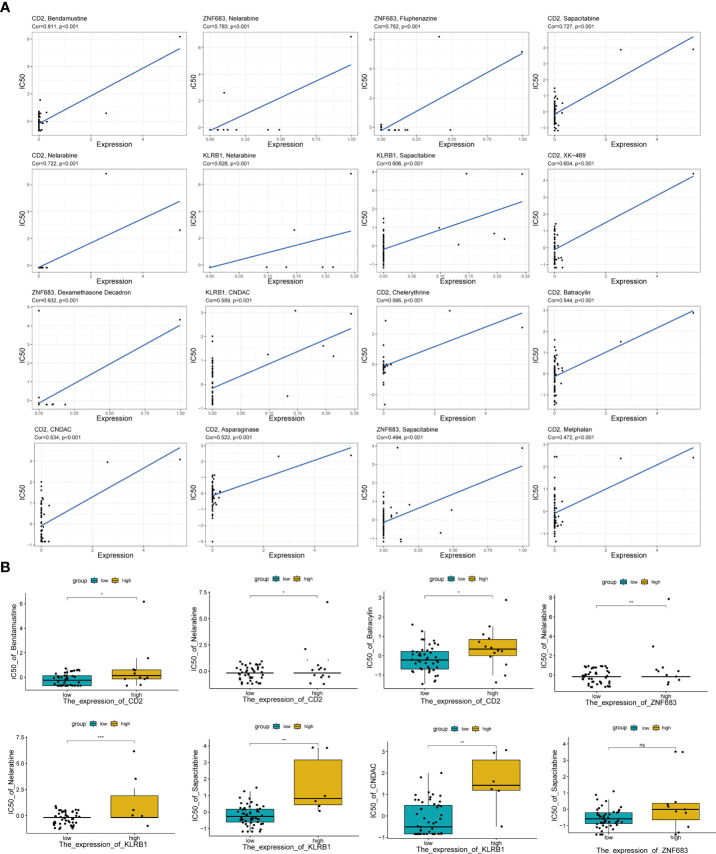
**(A)** The correlation analysis between expression levels of *CD2*, *ZNF683* and *KLRB1* with IC50 of anti-tumor drugs. **(B)** Boxplot showed IC50 difference of anti-tumor drugs in high-/low-expressed subgroups for *CD2*, *ZNF683* and *KLRB1*. IC50, the half maximal inhibitory concentration *p < 0.05, **p < 0.01, ***p < 0.001.

Moreover, to further estimate the influence of the risk score on drug sensitivity, we explored the correlation between the expression levels of Pyro-GPS and the response to chemotherapeutic drugs (IC50) in cancer cell lines. Significant positive correlations (p< 0.001) were observed in *CD2* with bendamustine, nelarabine, CNDAC, asparaginase, sapacitabine, chelerythrine, XK-469, batracylin and melphalan, in *ZNF683* with nelarabine, dexamethasone decadron, fluphenazine and sapacitabine, and in *KLRB1* with nelarabine, CNDAC and sapacitabine (**Figure 5A**). Then based on the median expression levels of *CD2*, *ZNF683* and *KLRB1* separately, the breast cancer patients were divided into high-expressed subgroups and low-expressed subgroups. Significant differences in IC50 were found in nelarabine (p< 0.001) and in sapacitabine and CNDAC (p< 0.01) between the *KLRB1* subgroups, in nelarabine between the *ZNF683* subgroups (p< 0.01), and in bendamustine, nelarabine and batracylin between the *CD2* subgroups (p< 0.05) (**Figure 5B**). The remaining results were shown in **Supplementary Figure 3A**. Moreover, the high-expressed subgroups were more associated with the high IC50 of drugs. Therefore, the risk score of Pyro-GPSs may be a potential biomarker to assess the efficacy of anti-tumor drugs and to choose appropriate treatment strategies.

### Stratification analysis and independent prognostic value of Pyro-GPS risk model

**Figure 6:**
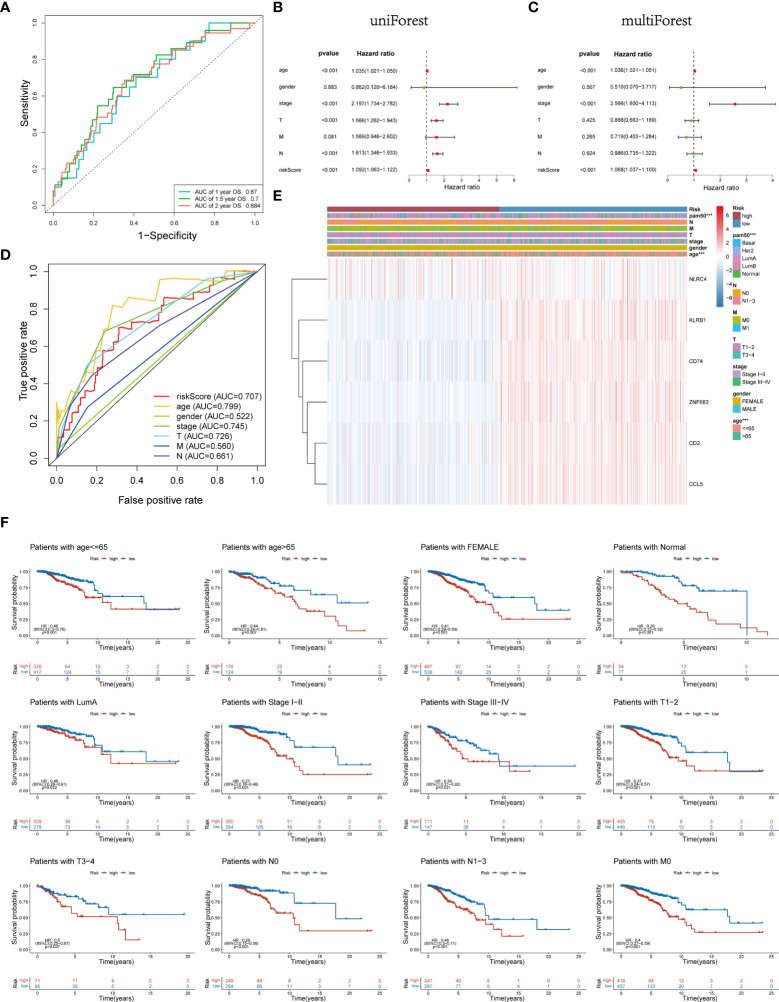
**(A)** Time-dependent ROC curve of 1, 1.5, and 2 years for survival prediction model. **(B, C)** Univariate and multivariate Cox regression analyses proved risk score was deemed to be an independent prognostic predictor. **(D)** ROC curves for the risk score, age, gender, stage, T, M and N. **(E)** Heatmap of the association between the expression levels of the six Pyro-GPS and clinicopathological features in TCGA dataset. **(F)** Stratification analysis based on clinicopathological features to compare survival difference in high-/low-risk subtypes. ROC, receiver operating characteristic; Pyro-GPS, pyroptosis-related genes prognostic signature; TCGA, The Cancer Genome Atlas; ***p < 0.001.

**Figure 7:**
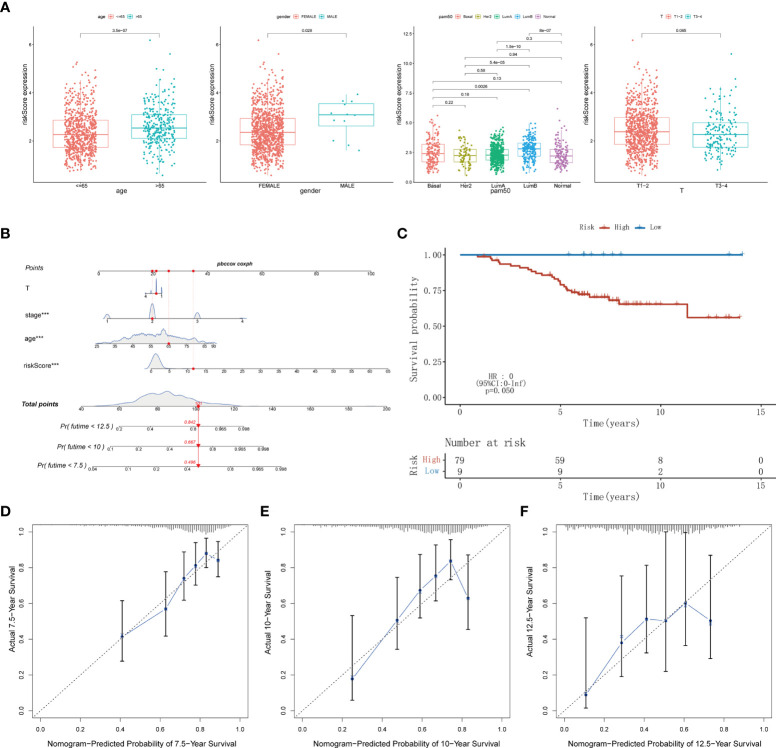
**(A)** The difference of risk score in clinicopathological classification including age, gender, PAM50 intrinsic subtypes and T stage. **(B)** Nomogram based on risk score, age, stage, T stage constructed to predict the 7.5-, 10-, and 12.5-year survival for BRCA patients. **(C)** Kaplan–Meier curves for BRCA patients to validate predictive value of Pyro-GPS risk model in the GSE20711 cohort. **(D-F)** Calibration plots of the nomogram for predicting the survival probability of OS at 7.5, 10, and 12.5 years. BRCA, breast cancer; Pyro-GPS, pyroptosis-related genes prognostic signature; ***p < 0.001.

Time-dependent ROC analysis of the TCGA cohort showed the prognostic model had high predictive efficiency (AUC= 0.670, 0.700, 0.684 for 1-, 1.5- and 2-year survival rates respectively) (**Figure 6A**). Next, univariate and multivariable Cox regression analyses demonstrated that the risk score derived from the Pyro-GPS model can serve as an independent prognostic factor for breast cancer patients in the TCGA cohort. The univariate Cox regression analysis indicates that the risk score is significantly associated with OS (HR: 1.092, 95%CI: 1.063–1.122, p< 0.001, **Figure 6B**). The multivariate Cox regression analysis proves that the risk score can independently predict poor survival (HR: 1.068, 95%CI: 1.037–1.100, p< 0.001, **Figure 6C**). Moreover, the ROC curve in **Figure 6D** illustrates that the AUC for the risk model is 0.707, which means the risk score has a satisfactory predictive efficacy. In addition, the connections between the risk groups and clinicopathologic features including age, gender, stage, T stage, M stage, N stage, and PAM50 intrinsic subtypes are diversely distributed in **Figure 6E**. Also, the heatmap demonstrates that the expression levels of *CD2*, *CCL5*, *KLRB1*, *CD74* and *ZNF683* decrease with the increasing risk score, while *NLRC4* is highly expressed in the high-risk group. Subsequently, the Kaplan–Meier curves in **Figure 6F** displays that the breast cancer patients with the following features possess survival advantage in the low-risk group: age ≤65, female, Normal, LumA, stage I–II, stage III–IV, T1–2, T3–4, N0, N1–3 and M0 subtypes. While patients with male, Basal, Her2, LumB, and M1 are not distinctly different between the low- and high-risk groups (**Supplementary Figure 3B**). Furthermore, we compared different expressions of risk score in diverse groups stratified by the above clinicopathological features. In brief, patients with age>65 or T1-2 stage or male have higher risk scores than patients with age ≤65 or T3–4 stage or female (**Figure 7A**). With regard to PAM50 molecular subtyping, the risk score expressions of Basal and LumB subtypes are distinctly upregulated compared with Her2, LumA and Normal subtypes. The rest data were shown in **Supplementary Figure 3C**.

### Construction of the Pyro-GPS-based nomogram

Herein, a nomogram (including several significative clinicopathological factors and risk score based on Pyro-GPS) was constructed to predict the 7.5-, 10-, and 12.5-year survival rates of the patients (**Figure 7B**). The predictive accuracy of the nomogram was assessed using calibration curves. The actual 7.5-, 10-, and 12.5-year survival probabilities of the breast cancer patients in the TCGA cohort are roughly consistent with the nomogram- predicted ones in **Figures 7D-F**.

### External validation of the Pyro-GPS in the GSE20711

Ultimately, an external validation cohort GSE20711 comprising 88 samples was assayed to validate the prognostic value of the Pyro-GPS in the breast cancer patients. The Kaplan–Meier survival curves in **Figure 7C** showed that patients in the high-risk group had poor prognosis than the low-risk group (p =0.050). This result is highly consistent with the findings of the TCGA cohort.

### Inhibition of MDA-MB-231 cells progression with *CCL5* upregulation

**Figure 8:**
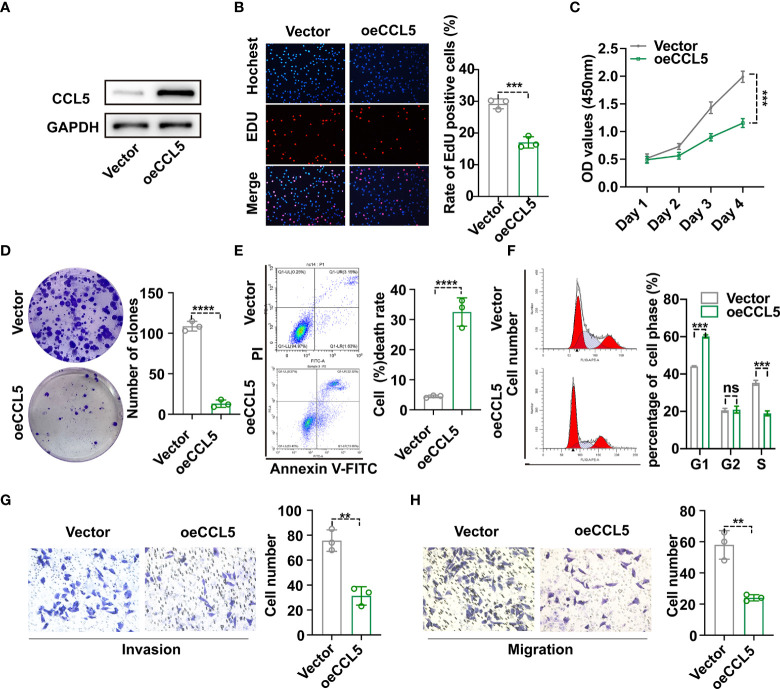
**(A)** Construction of *CCL5* over-expressed stable MDA-MB-231 cell line and verification *via* western blot analysis. The proliferation and clonogenic capacity of MDA-MB-231/oe-*CCL5* cells and MDA-MB-231/Vector cells were examined *via* EdU assay **(B)**, Cell Counting Kit-8 assay **(C)** and Colony formation assay **(D)**. Flow cytometric analysis combining with Annexin V-FITC/PI staining **(E)** and PI staining **(F)** were used to assess cell apoptosis rate and cell cycle arrest state. The effect of up-regulated *CCL5* on breast cancer cell invasion **(G)** and migration **(H)** based on transwell assays. oe-*CCL5* cells: *CCL5* over-expressed cells; **p < 0.01, ***p < 0.001, ****p < 0.0001.

To begin with, hub gene *CCL5* extracted from the protein interaction network emerged as the research object of cell experiments (**Supplementary Figure 4**). To investigate the effect of *CCL5* on occurrence and development of breast cancer, plasmid was transfected into MDA-MB-231 cell line to construct *CCL5* overexpression stable cell line, whose *CCL5* protein level was verified *via* western blot analysis in **Figure 8A**. In addition, EdU assay observed that overexpression of *CCL5* significantly reduced the EdU positive cells number of breast cancer cells (BC cells) (**Figure 8B**). The growth curves detected by CCK8 assay indicated that upregulation of *CCL5* could distinctly inhibit the proliferation of BC cells (**Figure 8C**). In accordance with the results of EdU assay and CCK8 assay, colony formation assay presented *CCL5*-amplifying group had prominently less cell clones number than control group in **Figure 8D**. Thus the outcomes of the above three *in vitro* experiments confirmed that overexpression of *CCL5* might restrain breast cancer growth potential and clone formation capacity. Furthermore, PI staining combining flow cytometry in **Figure 8F** displayed that upregulation of *CCL5* progressively increased the percentage of cells in the G1 phase to the detriment of the S phase. Annexin V-FITC/PI staining presented the proportion of apoptotic cells were dramatically increased by upregulating *CCL5* (**Figure 8E**). To some extent, apoptosis rate augment and cell cycle G1-phase arrest were two essential factors contributing to the growth suppression of MDA-MB-231 cells in the context of *CCL5* amplification. Subsequently, in order to assess the effect of magnifying *CCL5* on breast cancer metastatic ability, transwell assays with or without coating by matrigel were preformed to acquire functional significance of *CCL5* on MDA-MB-231 cells invasion and migration. Obviously, the quantities of cells arriving in lower chamber were much less in *CCL5* overexpression group than the control (**Figure 8G, H**). Consequently, our results of above cell experiments demonstrated that high expression level of *CCL5* might be correlated with poor prognosis as well as early recurrence in breast cancer patients.

## Discussion

As we all know, breast cancer is accompanied with high morbidity rate, and has become the first cause of cancer-associated death among women in 2020 according to the latest data (1). Breast cancer is a highly heterologous tumor characterized as diverse histogenesis and molecular pathogenesis (5, 27). Thus, gene expression, cell morphology, tumor microenvironment, drug sensitivity, metabolism, proliferation, migration and metastasis potential may all differ among the molecular types of breast cancer (28, 29). Moreover, triple negative breast cancer with poor prognosis has more prominent genomic instability and more significant immunogenicity (30, 31).

Pyroptosis is a novel form of programmed cell death featured by the fragmentation of cell membranes and the release of inflammatory factors (32). Increasing evidence implicates that pyroptosis can affect the tumorigenesis and progression of breast cancer, melanoma, colorectal cancer, gastric cancer, hepatocellular liver cancer, and lung cancer (33–37). As for breast cancer, pyroptosis reportedly plays a dual-role in tumor development and therapeutic mechanisms. The pyroptosis-related inflammasome and cytokine IL-1β contribute to angiogenesis and invasiveness of breast cancer, and a high inflammatory environment may be significantly associated with the high recurrence rate of breast cancer (38). To the contrary, gasderminB-mediated pyroptosis executed by natural killer cells and cytotoxic T lymphocytes enhances anti-tumor immune response (14). Moreover, GSDME (DFNA5) can be cleaved by granzyme B (GzmB) to activate pyroptosis and stimulate tumor-associated macrophages, tumor-infiltrating natural-killer and CD8+ T lymphocytes to evoke anti-tumor immunity in breast cancer (13). In addition, GSDME expression is suppressed in various cancers and its low level is correlated with poor survival of breast cancer patients, indicating GSDME may serve as a tumor suppressor (18). Therefore, there is an urgent need to identify the pyroptosis-related prognostic signatures to better help with accurate diagnosis and individualized treatment.

An initial objective of our project was to identify sensitive pyroptosis-related prognostic signatures and explore the underlying mechanisms how these prognostic biomarkers influence tumor progression through pyroptosis for breast cancer. Thus, data on 1089 breast cancer patients derived from TCGA were analyzed by a series of bioinformatics methods to achieve the research objective. First, 161 pyroptosis-related genes were extracted *via* literature search and Pearson’s correlation analysis. Then six superior Pyro-GPS were identified through univariate and multivariate Cox regression analyses, including two positive genes (*CD2* and *NLRC4*) and other four genes (*CCL5*, *KLRB1*, *CD74*, and *ZNF683*), in which the negative coefficients suggest higher expressions are correlated with better prognosis. The risk score of each sample was calculated *via* the expression levels of six Pyro-GPSs and their coefficient. Additionally, the 1089 samples downloaded from TCGA were randomly divided into a training cohort and a testing cohort. Then the two cohorts were separately stratified into a high-risk group and a low-risk group according to the median risk score. Results revealed that the low-risk group had survival advantage over the high-risk subgroup. The AUCs further confirmed the prognostic ability of the risk score. Moreover, the results of the external validation cohort obtained from GEO match with those observed in the above two cohorts on the predictive ability of these six signatures for breast cancer. Furthermore, cell proliferation assays including EdU, CCK8 and colony formation assay verified the essential role of CCL5 in inhibiting breast cancer cell growth and activity. Flow cytometry combined with special staining was utilized to confirm amplification of CCL5 could increase cell apoptosis and impede cells entering S phase. Cell invasion and migration experiments suggested that CCL5 essentially restrained the metastasis of breast cancer.

A significant negative correlation was observed between the risk score and survival time for the breast cancer patients. The greater absolute values of the coefficients with *NLRC4*, *KLRB1* and *ZNF683* represent a greater influence on risk scores than other three biomarkers. With obesity as a risk factor and associated with worse clinical outcomes for breast cancer, Ryan Kolb put forward that activation of the obesity-associated NLRC4 inflammasome/interleukin-1β signal pathway drives disease progression *via* the adipocyte-mediated vascular endothelial growth factor A expression and angiogenesis (39). Moreover, angiopoietin-like 4 reportedly plays a role in angiogenesis and breast cancer progression (40). These studies further confirm that *NLRC4* as a proto-oncogene can promote the progression of breast cancer.

Our study indicates that the low-risk patients with high expressions of protective Pyro-GPSs show higher immune cell infiltration and immune-oncology target expression, accompanied with better survival. This result means pyroptosis can enhance anti-tumor immune to prolong the survival time of cancer patients. This conclusion is consistent with other studies. A bioinformatics study reveals that the up-regulation of *KLRB1* (a TME-associated and immune-related signature) is correlated with favorable survival in breast cancer patients (41). Additionally, *KLRB1*(*CD161*) is linked to the pro-inflammatory functions of natural killer cells (42). These results raise the possibility that *KLRB1* may react on breast cancer through pyroptosis as a pro-inflammatory cell death. In addition, a review summarizes that tissue-resident memory (TRM) T cell as a subgroup of specific tumor-infiltrating lymphocyte is critical in preventing the proliferation and migration of solid tumors (43). A higher proportion of *ZNF683*-overexpressed TRM (*ZNF683*/*Hobit* is a characteristic gene of TRM) may become a better prognostic indicator and be associated with better immunotherapeutic response in lung cancer (44). Furthermore, high serum CCL5 level involved in cancer immune reactions is remarkably associated with longer disease-free survival and OS of patients with early breast cancer (45). Vps34 kinase inhibitor (Vps34i) can induce a T cell-inflamed tumor microenvironment construction (including infiltration of NK, CD8+, and CD4+ T effector cells) featured by high amplification of CCL5, CXCL10, and IFN-γ, thereby converting immune cold tumors (poorly infiltrated) into hot ones (highly infiltrated) (46). Moreover, Vps34i can be combined with anti-PD-L1/PD-1 immunotherapy to enhance antitumor efficacy in melanoma and CRC tumors (46). Thus, pyroptosis strongly related with TIME (including immune cell infiltration, levels of pro-inflammatory chemokines and cytokines) in tumorigenesis and development. After that, we explored the possible discrepancy of genetic variations for breast cancer patients in the two risk groups. Unique molecular biological characteristics of breast cancer, including intra-tumor heterogeneity, genomic instability and immunogenicity (30, 31, 47), prompt us to explore the genetic backgrounds on breast cancer. Results demonstrate that the high-risk patients have high mutation frequency of *TP53*, while the low-risk patients possess high mutation frequency of *PIK3CA*. However, no significant difference in variant classification, SNP/INS/DEL, SNV type and CNV exists between the two risk groups. *TP53* is a tumor suppressor involved in regulation of cell cycle, DNA damage repair, apoptosis, inflammmation and immune response (48). Much research points out that *TP53* mutations play a negative role in anti-tumor immunity and immunotherapy response, which is related to the poor prognosis in cancer patients (49). The results of the above studies are consistent with our result that the high-risk group gets poor clinical outcome.

Subsequently, GSEA and GSVA reveal that the low-risk group is closely associated with TIME and immune response, while the high-risk group is prominently associated with protein ubiquitination and deubiquitination. Ubiquitination as a reversible protein post-translational modification is a triple enzyme cascade reaction, which involves ubiquitin activation by E1 enzymes, ubiquitin conjugation to E2 enzymes, and ubiquitin ligation to the substrate protein *via* E3 enzymes. Studies prove that ubiquitination is involved in a plethora of physiological processes (e.g. cell cycle, cell death, transcriptional regulation, signal transmission, DNA damage repair and immune signaling) through regulating protein stability, localization, activity and interaction (50–54). A review in 2021 summarizes that ubiquitination can dynamically regulate inflammation and programmed cell death, which is deemed as the crucial components of TNF-stimulated cell death and NLRP3 inflammasome–mediated signaling (55). Besides, a distinct connection between TRAF3 (tumor necrosis factor receptor-associated factor 3)-mediated ULK1 (Unc-51 like autophagy activating kinase) ubiquitination in macrophages and pyroptosis was found (56, 57). Reportedly, human papillomavirus E7 can recruit E3 ligase TRIM21 to induce degradation and ubiquitination of IFI16 inflammasome, resulting in suppression of cell pyroptosis and occurrence of immune escape (58). In addition, aberrant ubiquitin regulation of inflammatory pathways can induce the onset and progression of cancers and autoinflammatory diseases, and thus targeting dysfunctional ubiquitination may be a promising treatment strategy (55). For instance, downregulation of deubiquitinating enzyme USP47 is associated with shorter disease-free survival of colorectal cancer (CRC) patients, and USP47-mediated deubiquitination of transcription elongation factor a3 can inhibit pyroptosis and apoptosis of CRC cells treated with chemotherapeutic doxorubicin, which may be a target for therapeutic intervention in CRC (59).

Furthermore, the tumor functional patterns including the Wnt and the TGF-β signaling pathways are enriched in the high-risk group. A review in 2020 reveals that Wnt signaling plays a crucial role in the proliferation, metastasis, TIME regulation, therapeutic resistance, phenotype shaping and stemness maintenance of breast cancer (60). Interestingly, most Wnt signaling factors such as β-catenin, Axin, GSK3, and Dvl are regulated by ubiquitination and deubiquitination, and the inhibitors of deubiquitinating enzymes may be applied for cancer therapeutic strategies (61). In addition, transforming growth factor (TGF)-β was originally deemed to be a potent proliferation inhibitor and apoptosis inducer in early breast cancer, but was later proved to increase cancer progression in the advanced stages (62). Notably, TGF-β can attenuate immune response, including tumor immune evasion and poor responses to cancer immunotherapy, *via* influencing diverse immune cells in the tumor microenvironment (63). Given the existing findings, intricate connection may exist among pyroptosis, ubiquitination and TIME to affect tumor proliferation, invasion and migration.

Additionally, IC50 of the mentioned anti-cancer drugs, especially Nelarabine, is highly positively correlated with the expressions of *CD2*, *ZNF683* and *KLRB1*, which means drug resistance. These results point out that the expression levels of these genes can affect drug response and may be potential biomarkers for establishing appropriate treatment strategies. After that, our univariate and multivariate Cox regression analyses indicate risk score can serve as an independent prognostic factor for breast cancer patients. Then a nomogram model combining six pyroptosis-related genes and other clinicopathological features was constructed and used as an applicable quantitative tool to predict the survival of breast cancer patients. Undeniably, several limitations still exist in our study. Firstly, due to the lack of available clinical data in our center, no further external verification of Pyro-GPS can be performed. Secondly, how pyroptosis-related genes affect the prognosis of breast cancer patients *via* regulating the TIME is still indistinct and needs further exploration. Next, the association between high risk score and protein ubiquitination lacks experimental validation. In addition, only three Pyro-GPSs were included to analyze their connections with anti-tumor drugs response for breast cancer because of insufficient database information.

On the whole, we established a pyroptosis-related gene prognostic model and assessed the discrepancy in tumor immune microenvironment, gene mutation landscape and enrichment pathways between the two risk groups for breast cancer. Meanwhile, we verified that the hub gene *CCL5* could inhibit the proliferation, invasion and migration of BC cells as well as promote BC cells apoptosis. These signatures may serve as potential targets to predict survival and develop treatment strategies for breast cancer patients.

## Data availability statement

The original contributions presented in the study are included in the article/**Supplementary Material**. Further inquiries can be directed to the corresponding authors.

## Author contributions

HZ, XY and JY: designed the study and drafted the manuscript. GH and XZ: analysis and interpretation of the data. XW: acquisition of the data. LS and YZhou: checked the figures. XC: typeset article format. XL and YZhu: revised the manuscript critically for important intellectual content. All authors contributed to the article and approved the submitted version.

## Funding

This study was funded by the National Natural Science Foundation of China (Grant Nos. 82072931 and 82002805).

## Conflict of interest

The authors declare that the research was conducted in the absence of any commercial or financial relationships that could be construed as a potential conflict of interest.

## Publisher’s note

All claims expressed in this article are solely those of the authors and do not necessarily represent those of their affiliated organizations, or those of the publisher, the editors and the reviewers. Any product that may be evaluated in this article, or claim that may be made by its manufacturer, is not guaranteed or endorsed by the publisher.
