# Supplementary figures and images for: Comprehensive analysis of pyroptotic gene prognostic signatures associated with tumor immune microenvironment and genomic mutation in breast cancer
Source: Front Immunol. 2022 Aug 25;13:933779. doi: 10.3389/fimmu.2022.933779 (PMC9453314; doi:10.3389/fimmu.2022.933779)

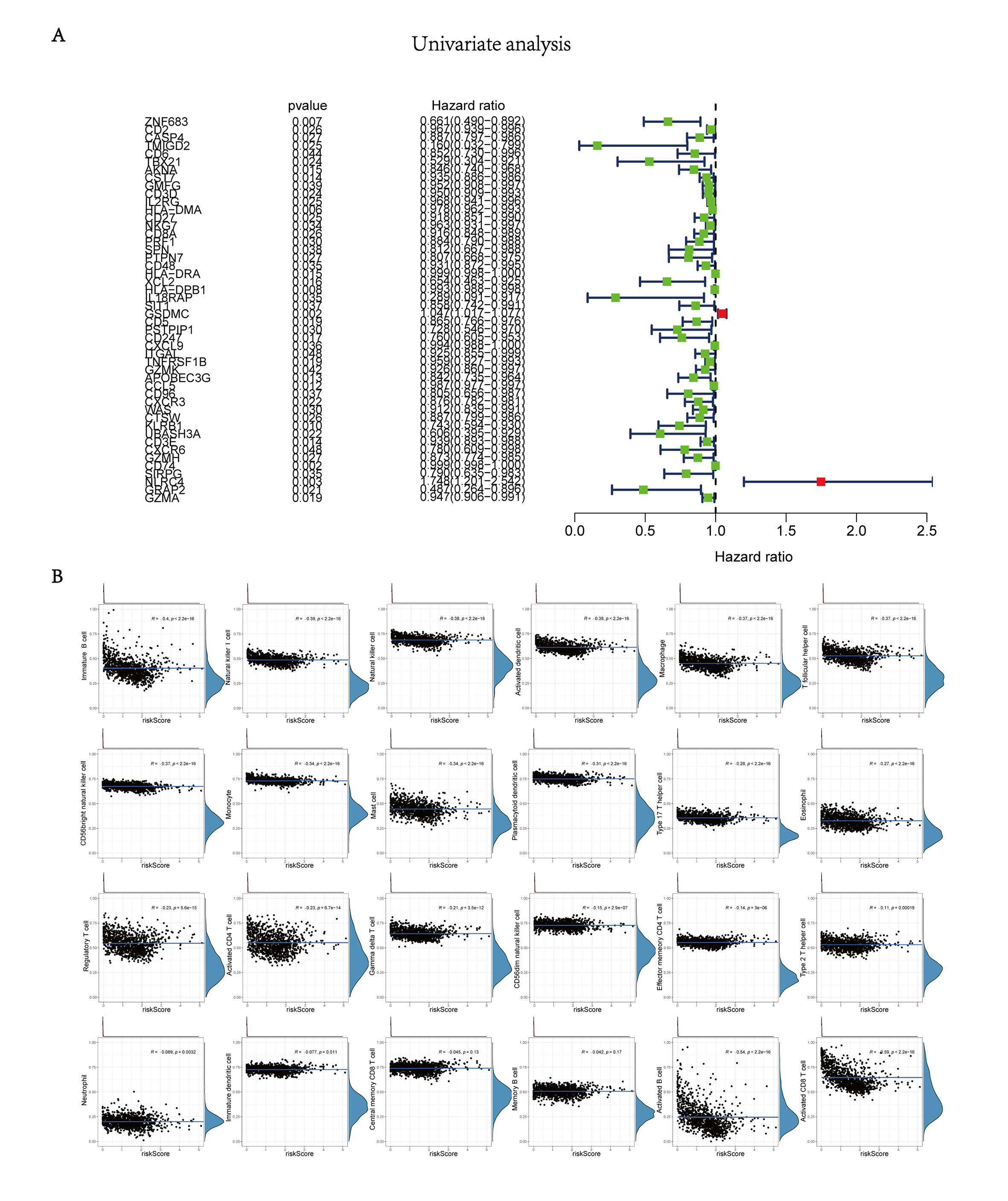

Supplement: Supplementary file 1 [file Image_1.tif]

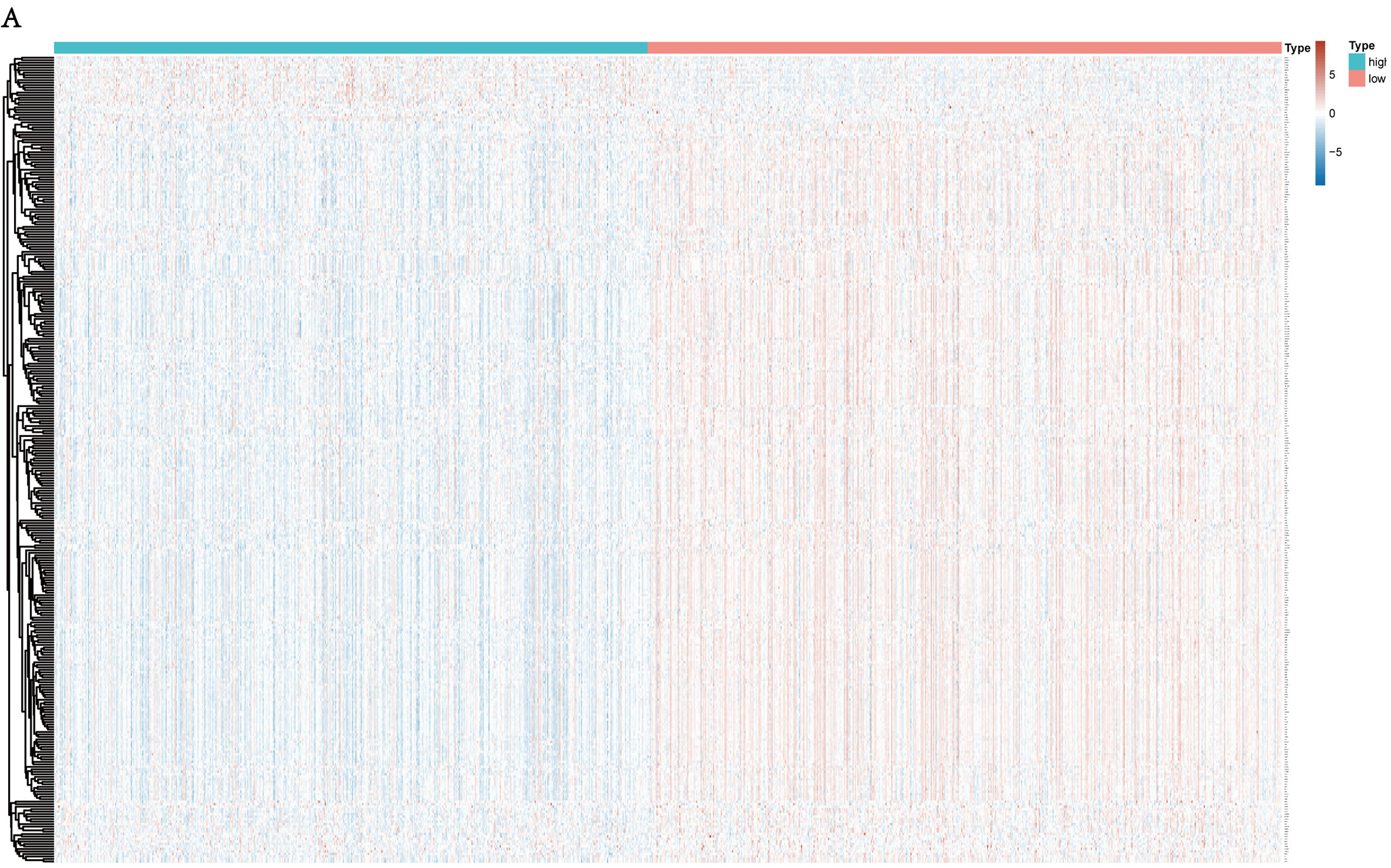

Supplement: Supplementary file 2 [file Image_2.tif]

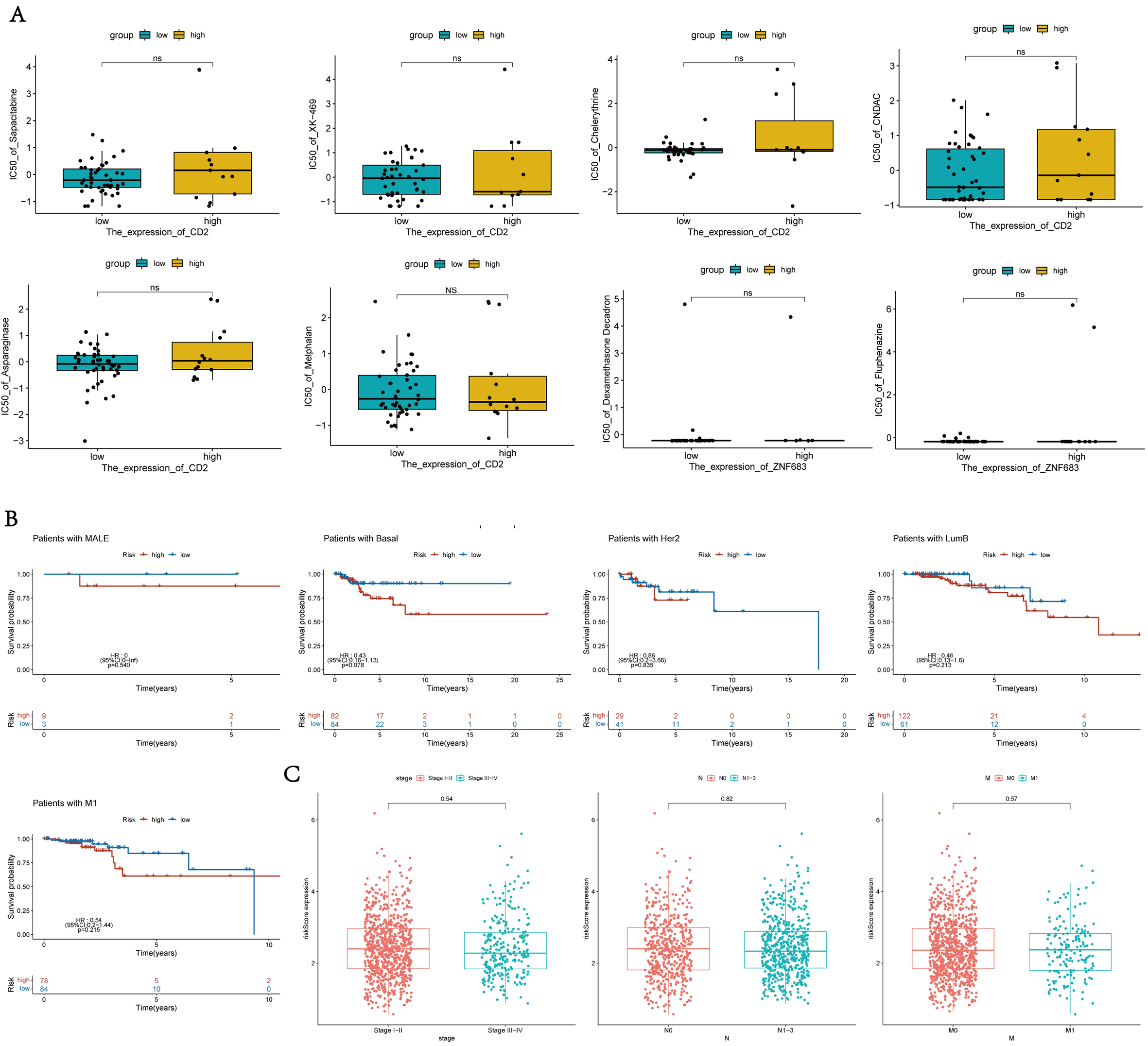

Supplement: Supplementary file 3 [file Image_3.tif]

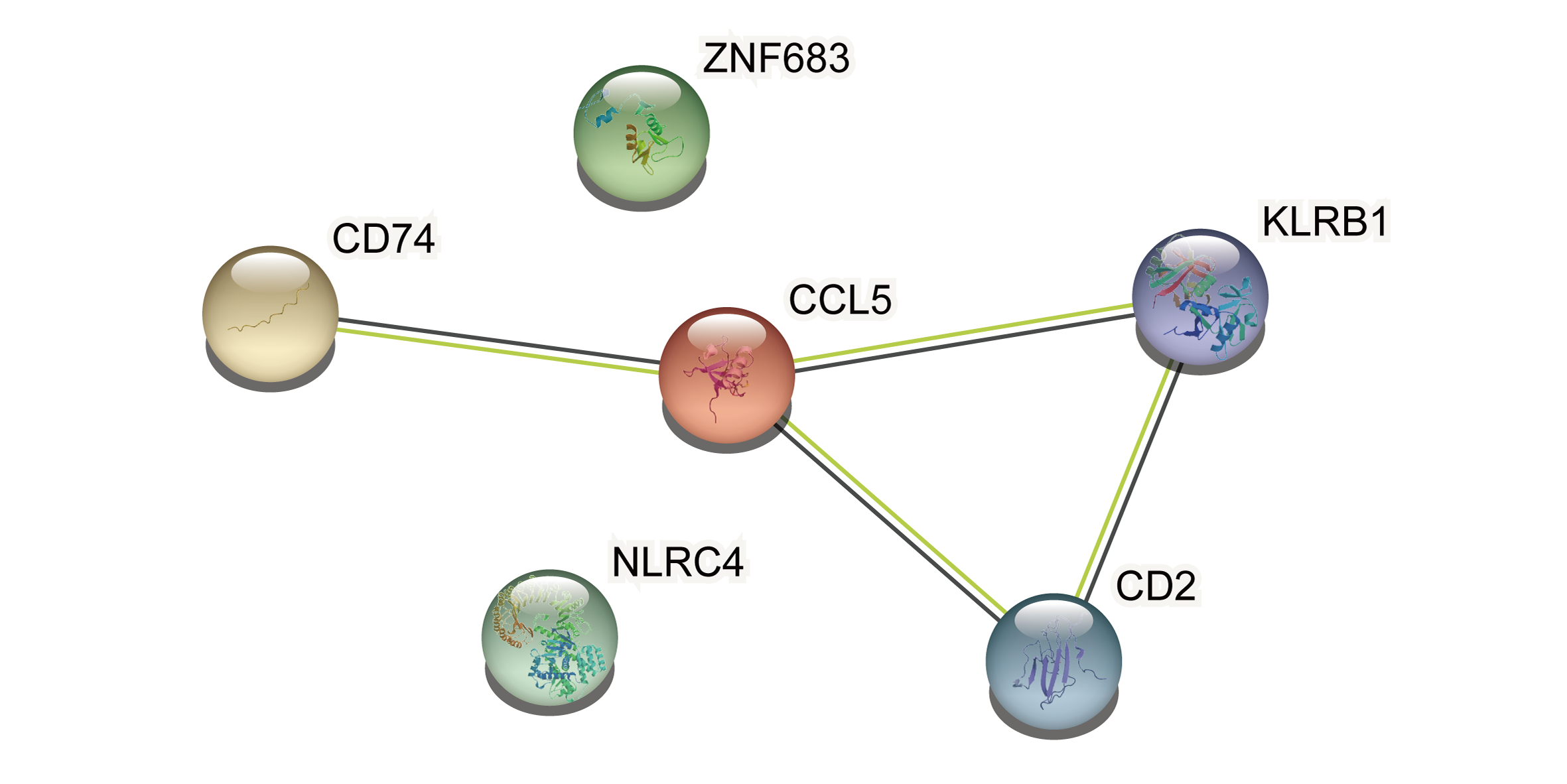

Supplement: Supplementary file 4 [file Image_4.tif]
